# The Psychological Effects of a Campus Forest Therapy Program

**DOI:** 10.3390/ijerph17103409

**Published:** 2020-05-14

**Authors:** Jin Gun Kim, Tae Gyu Khil, Youngsuwn Lim, Kyungja Park, Minja Shin, Won Sop Shin

**Affiliations:** 1 Department of Forest Therapy, Chungbuk National University, Cheongju 28644, Korea; jingun0308@naver.com (J.G.K.); ktg0704@hanmail.net (T.G.K.); suwnmail@naver.com (Y.L.); parkaous@hanmail.net (K.P.); yeamolove@hanmail.net (M.S.); 2 Department of Forest Sciences, Chungbuk National University, Cheongju 28644, Korea

**Keywords:** forest healing, campus forest, profile of mood state, stress response inventory, university students’ stress

## Abstract

This study aimed to examine the psychological effects of a campus forest therapy program. To evaluate these, pre-test and post-test control group design was employed. A total of 38 participants participated in this study (19 in the campus forest therapy program group, and 19 in control). The Profile of Mood State (POMS) questionnaire and Modified form of the Stress Response Inventory (SRI-MF) were administered to each participant to assess psychological effects. The results of this study revealed that participants in the campus forest therapy program group had significantly positive increases in their mood and stress response compared with those of control group participants. In conclusion, the campus forest therapy program is an efficient strategy to provide psychological health benefits to university students and our study can inform decision-makers on the priority of the campus forest program in societal efforts to promote psychological well-being among university students.

## 1. Introduction

Psychological health problems among university students is an important topic. University students face many of the stressors, including academic demands, social challenges, and uncertainty about the future, which are linked to increased levels of stress and psychological health problems [1,2]. Regehr et al. [3] reported that more than 50% of college students experience significant levels of anxiety and depression. The American College Health Association [4], also reported in study results using 80,000 college students that 62% of students suffered overwhelming levels of anxiety and 40% of the students suffered depression.

The college students with psychological health problems reported negative academic impact [5,6], relationship dysfunction [7], high rate of drinking [8,9] and substance use [10,11,12], and high incidence of suicide [13]. College is an important time, in which young people can adopt lasting healthy lifestyle habits but is associated with increased chronic disease risk [14,15]. In the absence of a healthy means to cope or proper support networks, this increase in psychological problems can be extremely taxing on the body. It is therefore not surprising that maladaptive coping strategies and unhealthy lifestyle choices are both prevalent and problematic in this population [16]. Therefore, it is important to cope with psychological problems during the critical university stage. The use of forest and forest therapy is increasingly recognized as an effective intervention for dealing with psychological problems [17,18]. Numerous studies have demonstrated the effects of using the forest in relieving stress levels and inducing psychological relaxation [19,20,21,22]. Spending time in a forest environment has also been shown to enhance immune function by promoting the activity of Natural Killer (NK) cells [23], to reduce the stress hormone cortisol concentration [24,25]. Visiting the forest is also increasingly recognized for its potential to manage stress, and to promote mental, psychological, and physical health. Based on the empirical research evidence, The Korea Forest Service (KFS) facilitated “Forest Therapy” to utilize forests for enhancing people’s health and quality of life. The KFS has legitimized the concept of forest therapy and launched a forest therapist system to develop and manage forest therapy programs [26].

“Forest therapy program” is a set of structured activities and cognitive-behavioral therapy-based interventions using various elements of the forest environment to mitigate stress and to promote health [27]. Regarding the psychological effectiveness of forest therapy programs, many previous studies have reported improvement in depression, self-esteem, and anxiety [28,29,30,31]. For example, Jang et al. [32] also reported that an after-school forest therapy program for infant participants was effective in improving pro-social behavior and efficiency of expressing themselves. For the office workers, Shin et al. [33] reported that the two-day forest therapy program provided significantly positive changes in workers’ job stress and moods.

The campus forest represents a preexisting, accessible, and effective resource for enhancing psychological health [34]. For example, Lee and Shin [35] reported that the forest therapy program, performed at a university campus forest using university students, provided significantly positive emotional improvement. Using 558 voluntary college students, Ibes et al. [34] also reported that the campus forest provided a significant psychological impact on students, most commonly relief from stress. Bang et al. [36] conducted a campus forest-walking program targeting university and graduate students during their lunchtime and reported improvement in participants’ depression and physical function.

To date, many empirical research results show that forest therapy programs provide a wide range of psychological health benefits to the program participants [37,38,39]. However, few studies on the psychological effects of campus forest therapy have yet been reported. Therefore, this study was conducted to verify the psychological effects of campus forest therapy programs for university students and to provide basic data for the development of various forest therapy programs for university students in the future. Through this study, we hope to inform decision-makers on the priority of the campus forest program in societal efforts to promote psychological well-being among university students.

## 2. Materials and Methods

### 2.1. Participants

The participants in this study were 38 university students, 24 males (63%) and 14 females (37%), with a mean age of 22 years. Recruitment posters were posted throughout the university buildings to recruit volunteers. No incentives were provided to the volunteers. The inclusion criteria required the participants to be current students at the specified university. More than that, participants who met the following inclusion and exclusion criteria were considered eligible for the study: (1) no diagnosis of reaction to severe stress and/or a depressive episode; and (2) could not be suffering from any drug or alcohol abuse.

We employed the “pretest-posttest control group design” and used a control group because it is practically impossible to eliminate all of the bias and outside influence that could alter the results of the experiment. To secure homogeneity between the forest therapy program and the control group, the participants were randomly distributed into the two groups (i.e., 19 campus forest therapy program group and 19 control group).

The experiment was conducted during the second semester of 2019 (September–November). A total of eight sessions’ campus forest therapy program delivered by therapists was performed. The participants were fully informed about the study’s purpose and signed an agreement. This study was approved by the Institutional Review Board of Chungbuk National University (IRB number: CBNU-201910-SB-945-01).

### 2.2. Experimental Sites

The field experiment was conducted in the Chungbuk National University campus forest in Korea. The dominant species in the campus forest are Metasequoia (*Metasequoia glyptostroboides*), Cypress (*Chamaecyparis pisifera*), and other broad-leaved tree species. The study area was a suitable place for conducting a forest therapy program in terms of accessibility, distribution of a variety of vegetation, and a low lope of topography. During the eight-sessions, the weather was clear, and the mean temperature was 16.2 °C ± 1.3 °C.

### 2.3. Experimental Design

#### Forest Therapy Program

**Figure 1:**
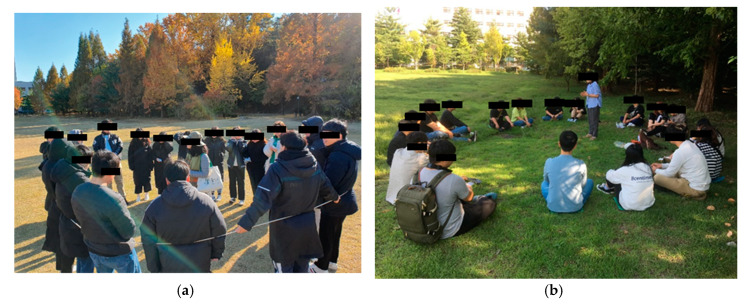
Campus forest therapy program intervention. (**a**) forest folk dance; (**b**) lecture on stress management.

To collect data, an eight-session forest therapy program was performed. Once a week, each session was delivered by a trained forest therapist for one and a half hours. During the eight sessions of the program, participants were involved in many forest therapy activities such as forest dance, forest meditation, forest exercise, walking, and others under the instruction of the therapist (see Table 1). The forest therapy activities were developed and distributed according to each appropriate sessions’ theme based on consultation with researchers in forest therapy/forest recreation and forest therapists (see Figure 1). The main theme of the program was to reduce stress and improve self-esteem for the participants. The participants in the control group did not receive leaflets, lectures, or any forest therapy activities and were asked to follow their routine activities during the experimental period.

### 2.4. Psychological Measurement

To measure the psychological effects of the experiments, three scales were administered to each participant before and after the intervention.

To assess the effect of forest therapy on emotional state, the Korean version of the Profile of Mood States (POMS) was employed [40]. The POMS is a reliable and valid instrument for assessing psychological distress [41,42] and has been used previously to estimate the influence of brief forest experience [19,20,21] as well as long-term forest therapy program on mood states [43,44]. The POMS measures six mood states: “tension–anxiety (T–A)” “depression-dejection (D)”, “anger–hostility (A–H)” “fatigue (F)”,’ “confusion (C)”, and “vigor (V)” [41,42]. A five-point Likert scale (0 = strongly agree to 4 = strongly disagree) was used for each item to evaluate each participant’s mood states. The Korean version of POMS has a relatively high reliability (Cronbach’s α = 0.85) [45].

To measure participant’s stress response levels, The Modified form of the Stress Response Inventory (SRI-MF) was used. The SRI-MF was originally developed by Koh et al. [46] and revised by Choi et al. [47]. The SRI-MF is a key measurement tool with respect to stress, particularly the mental health and physical symptoms related to stress [48] and has been used previously to estimate the effect of forest therapy program on stress [49]. The scale assesses participant’s stress response levels including somatization, anger, and depression. The SRI-MF has a total of 22 items, and each item has a 5-point Likert scale (1 = strongly disagree; 5 = strongly agree). The SRI-MF has relatively high reliability (Cronbach’s α = 0.93) [47].

### 2.5. Data Analysis

The data collected for this study were analyzed using SPSS 18.0 Windows (SPSS, Chicago, IL, USA). Descriptive statistics comprised means, standard deviation, frequency, and percentage to present socio-demographic information and outcome variables. The paired t-tests were conducted to compare participants’ psychological effects between pre- and post-tests for each group (forest therapy program and control groups). All statistical tests were used at a *p*-value of < 0.05 significance level.

## 3. Results

### 3.1. Profile of Mood States (POMS)

**Figure 2:**
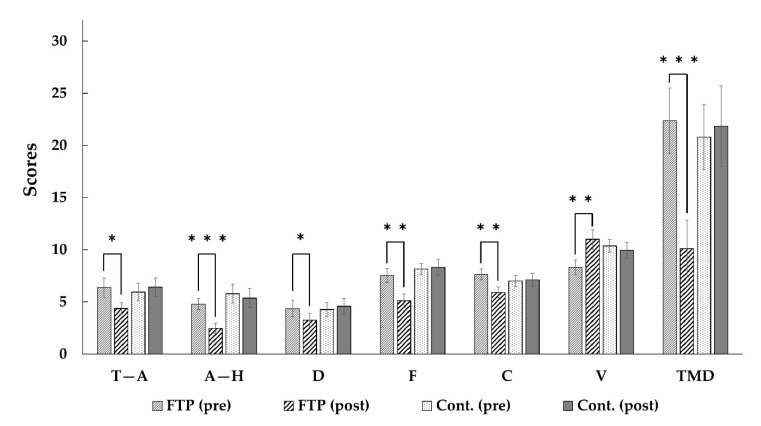
The results of paired *t*-test analyses of Profile of Mood States (POMS) scores. T–A = tension–anxiety; C = confusion; A–H = anger–hostility; D = depression; F = fatigue; V = vigor; TMD = Total Mood Disturbance. FTP = Forest Therapy Program Group; Cont. = Control Group. *** *p* < 0.001, ** *p* < 0.01, * *p* < 0.05.

The results of paired *t*-tests between pre- and post-tests POMS scores for each group are presented in Figure 2. As can be seen, there was a significant decrease in Total Mood Disturbance scores for campus forest therapy group after eight sessions of forest therapy program intervention (pre 22.37 ± 3.13, post 10.11 ± 2.73, t = 4.368, *p* = 0.000). The results of paired *t*-tests indicated that there were significantly positive changes in six sub-scales of the POMS: “tension-anxiety (pre 6.37 ± 0.92, post 4.37 ± 0.54, *t* = 2.387, *p* = 0.028)”, “anger-hostility (pre 4.79 ± 0.55, post 2.47 ± 0.49, t = 4.238, *p* = 0.000)”, “depression-dejection (pre 4.37 ± 0.79, post 3.26 ± 0.65, t = 2.207, *p* = 0.041)”, “fatigue-inertia (pre 7.53 ± 0.68, post 5.11 ± 0.64, t = 3.028, *p* = 0.007)”, “confusion-bewilderment (pre 7.63 ± 0.52, post 5.90 ± 0.52, t = 3.067, *p* = 0.007)”, and “vigor (pre 8.32 ± 0.71, post 11.00 ± 0.94, t = −2.661, *p* = 0.016)”.

However, no significant changes were found in the control group in Total Mood Disturbance (pre 20.79 ± 3.12, post 21.84 ± 3.85, t = −0.355, *p* = 0.726), nor in all six sub-scales of the POMS: “tension-anxiety (pre 5.95 ± 0.84, post 6.42 ± 0.87, t = −0.590, *p* = 0.563)”, “anger-hostility (pre 5.79 ± 0.90, post 5.37 ± 0.92, t = 0.671, *p* = 0.511)”, “depression-dejection (pre 4.26 ± 0.68, post 4.58 ± 0.74, t = −0.480, *p* = 0.637)”, “fatigue-inertia (pre 8.16 ± 0.53, post 8.32 ± 0.77, t = −0.221, *p* = 0.828)”, “confusion-bewilderment (pre 7.00 ± 0.54, post 7.11 ± 0.60, t = −0.244, *p* = 0.810)”, and “Vigor (pre 10.37 ± 0.60, post 9.95 ± 0.76, t = 0.522, *p* = 0.608)”.

To test equivalence of participants for forest therapy and control groups, we performed *t*-tests between the two groups’ pre-test scores. There were no significant differences in pre-test scores between the groups, except for “vigor” sub-scale (t = −2.212; *p* = 0.033). In order to support statistical validity, we evaluated reliability of this sub-scale. Cronbach alpha of 0.813 for this sub-scale indicated relatively high reliability.

### 3.2. Modified Form of the Stress Response Inventory (SRI-MF)

To evaluate the effectiveness of the participants’ stress response after the eight sessions of campus forest therapy experiences, a set of *t*-tests were performed with pre- and post-test SRI-MF scores.

**Figure 3:**
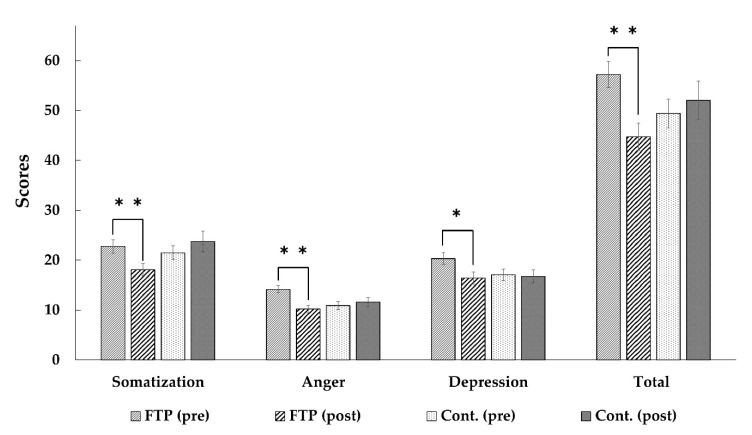
The results of paired *t*-test analyses of Modified Form of the stress response Inventory (SRI-MF) scores. FTP = Forest Therapy Program Group; Cont. = Control Group. ** *p* < 0.01, * *p* < 0.05.

For the participants in campus forest therapy program group, there were significant decreases in their total stress responses (pre 57.21 ± 2.65, post 44.74 ± 2.76, t = 3.690, *p* = 0.002), and all other sub-scales of the SRI-MF: “somatization (pre 22.74 ± 1.33, post 18.11 ± 1.21, t = 2.903, *p* = 0.009)”, “anger (pre 14.16 ± 0.70, post 10.21 ± 0.79, t = 3.980, *p* = 0.001)”, and “depression (pre 20.32 ± 1.21, post 16.42 ± 1.20, t = 2.866, *p* = 0.010)” (See Figure 3).

However, no significant changes were found in the control group participants’ total stress responses (pre 49.42 ± 2.90, post 52.05 ± 3.90, t = −0.795, *p* = 0.437), and all other sub-scales of the SRI-MF: “somatization (pre 21.47 ± 1.37, post 23.74 ± 2.07, t= −1.320, *p* = 0.203)”, “anger (pre 10.89 ± 0.78, post 11.58 ± 0.88, t = −0.646, *p* = 0.527)”, and “depression (pre 17.05 ± 1.19, post 16.74 ± 1.33, t = 0.317, *p* = 0.755)”.

To test equivalence of participants for forest therapy and control groups, we performed *t*-tests between the two groups’ pre-test scores. There were no significant differences in pre-test scores between the groups, except on “anger” sub-scale (t = −3.119; *p* = 0.004). In order to support statistical validity, we evaluated reliability of this sub-scale. Cronbach alpha of 0.730 for this sub-scale indicated relatively high reliability.

## 4. Discussion

This study evaluated the psychological effectiveness, especially regarding emotion and stress, of the campus forest therapy program. It revealed that participants in campus forest therapy intervention had significantly positive changes in their mood states and stress responses after the intervention. The result of this study indicated that campus forest therapy provides improvements in participants’ psychological health. The results of this study also provide a rationale to use campus forests to promote psychological well-being among university students.

The results of this study stand in the same line of an extensive number of studies providing evidence for and support of Attention Restoration Theory (ART). ART proposes that exposure to nature, such as forests, reduces mental fatigue or psychological stress. A theory attempting to explain these effects was proposed by Kaplan [50]. According to Kaplan’s ART, prolonged use of directed attention leads to fatigue of neural mechanisms. The recovery of effective functioning is enabled by settings that have certain key properties, such as “being away”, “extent”, “fascination”, and “comparability”. These components refer to those key properties of forests that trigger psychological states contributing to restorative experience [50,51]. These studies include those of Kaplan [50] and Song et al. [52]. The results of this study also support biophilia [53] and human evolutionary theories [54]; humans have spent many thousands of years adapting to the natural environment, yet have only inhabited urban ones for relatively few generations [55]. Evolutionary perspectives premise that, because humans evolved over millions of years in natural environments, we are to some degree physiologically and psychologically adapted to nature rather than to urban settings.

The rapid increase in the urban population worldwide is one of the important global health issues of the 21st century. According to the projections of the United Nations Population Division, by 2030 more people in the developing world will live in urban than rural areas; by 2050, two-thirds of its population is likely to be urban [56]. Urban dwellers face stressful situations in their living environments, such as work, school, and even home. Therefore, restoration from everyday stress is essential for their healthy lives. Therefore, the psychological benefits of forest therapy through campus forests are important, and the campus forest, which is easily accessible to students, is expected to have very important roles in promoting psychological health.

The results of this study revealed that the eight sessions of campus forest therapy program provided significant positive changes in the participants’ total mood, and other mood states such as “anger-hostility”, “tension-anxiety”, “depression-dejection”, “fatigue-inertia”, “confusion-bewilderment” and “vigor”. The results of this study are consistent with previous findings using a diverse population of participants such as middle-aged males [37], adults [39], senior citizens [57], and mental hospital patients with affective and psychotic disorders [58].

The advantage of explicitly studying forest induced mood is that mood has relevant and long-lasting consequences on such things as the immune system, physiological responses to the stressor, cognitive skills, and helping behavior. Thus, forest therapy and its consequent moods should be considered as socially relevant and deserving of public attention, especially for university students who suffer and face many stressors, including academic demands, social challenges, and uncertainty about the future. A forest therapy program using campus forests would be an effective and economic strategy, in terms of time and money, to cope with such stressors for university students.

Positive changes in university students’ mood states provide benefits beyond “feeling good”. According to Izard [59], mood state influences what is attended to in the environment and therefore can have a profound impact on subsequent cognition and behavior. Mood change, and mood in general, have physiological correlates. Mood is an integral part of many forest therapy studies [37,39,57,58] and is likely to be a product of forest therapy experiences. The significance of mood was demonstrated by noting the impacts of mood on cognition, behavior, and physiology These impacts include learning, task performing, helping behavior, socialization, and health [60]. The benefits resulting from improved mood induced by forest therapy experiences may be one of the major justifications to the university for the expenditure of its resources on the provision and management of campus forests.

This study also found that the eight sessions of campus forest therapy program provided a significant decrease in total stress responses, and other sub-scales of the SRI-MF such as “somatization”, “anger”, and “depression”. This study also confirms the results of previous empirical studies indicating the forest therapy programs’ effectiveness on stress reduction and coping with stress/stress response [61,62,63,64,65,66,67,68]. For example, Song et al. [65] reported forest therapy was effective for female nursing students’ stress reduction. Using participants who were office workers [64], middle-aged population [49], elementary students [66], and cancer patients [67], most of the studies have reported participants’ stress level decreases after taking forest therapy programs. Findings from scores of studies indicate that various stress mitigation benefits are consistently rated by forest therapy as very important consequences of their participation. The stress response is the process whereby a person responds physiologically, psychologically, and often with specific behaviors, to a situation that threatens well-being and health [68].

However, the study had several limitations. Firstly, the participants for this study were limited to healthy university students in their 20 s. To generalize the findings, further studies are needed using different groups of the population with different socio-demographic characteristics. Secondly, this study was conducted in a campus forest to validate the psychological effect of forest therapy. Effects according to the various characteristics of the greenspace must be examined in the future. Thirdly, participants’ prior expectations, preferences for nature and experiences of forest may influence the results. Fourth, in this study the control group conducted their usual activities. Some of the participants in the control group may use forests for their leisure, and those experiences may influence the results of this study. Therefore, further studies are needed with participants who spend time in forests without giving them any instructions. In sub-scales of “vigor” (Figure 2), and “anger” (Figure 3), there were significant differences in pre-test scores between the forest therapy and control groups. The differences indicated that the participants in both groups had different baselines of “vigor” and “anger” levels. These differences may influence the results of this study. Lastly, we recruited participants for this study as volunteers, using so called self-selection. So self-selection might influence the results of this study for generalizability. These limitations should be considered in future research. Despite these limitations, this study provides notable evidence of the effect of forest therapy in a campus forest, which is easily accessible to students.

## 5. Conclusions

This study showed that campus forest therapy intervention provided significant psychological effects. More specifically, there were significant positive changes in participants’ emotional states and stress responses. These results of study indicated the campus forest therapy program as a strategy to promote student’s mental health, thereby the effectiveness of forest therapy is suggested as a complementary therapy in modern urbanized society.

## Figures and Tables

**Table 1:** Themes and Activities of the Forest Therapy Program.

Theme	Session	Program Activities
Rapport building	1	Ice breaking introduction; familiarity with forest; lecture on stress management
	2	Clapping exercise; Forest folk dance
Stress reduction	3	Forest orienteering (using natural objects to solve group mission); Physical stimulation for relaxation
	4	Group gaming activities using natural objects (drawing natural objects, hit the target with an acorn)
Improvement of sense of belonging and self-esteem	5	Forest exercise (Forest walking, stretching)
	6	Barefoot walking in forest; Talking to Nature
Cooperation and trust	7	Natural object five senses game; Photo healing (taking pictures of nature and story-telling)
	8	Forest band exercise; rope game
